# Hepatitis A virus infections, immunisations and demographic determinants in children and adolescents, Germany

**DOI:** 10.1038/s41598-018-34927-1

**Published:** 2018-11-12

**Authors:** Kai Michaelis, Christina Poethko-Müller, Ronny Kuhnert, Klaus Stark, Mirko Faber

**Affiliations:** 1 Department for Infectious Disease Epidemiology, Unit of Gastrointestinal Infections, Zoonoses, and Tropical Infections, Robert Koch Institute (RKI), D-13353 Berlin, Germany; 2 Department of Epidemiology and Health Monitoring, Robert Koch Institute (RKI), D-12101 Berlin, Germany

## Abstract

Hepatitis A is a vaccine-preventable disease with a global distribution. It predominantly occurs in regions with inadequate living conditions, but also affects populations in industrialised countries. Children are frequently involved in the transmission of hepatitis A virus (HAV) and thus play a central role in the epidemiology of hepatitis A. Here, we investigated HAV infections, immunisations, and associated demographic determinants in a nationwide, population-based, cross-sectional survey conducted in Germany from 2003–2006. Out of 17,640 children and adolescents, complete data sets (HAV serology, demographic information and vaccination card) were available for 12,249 (69%), all aged 3–17 years. We found protective antibody levels (>=20 IU/L) in 1,755 (14%) individuals, 1,395 (11%) were vaccinated against hepatitis A, 360 (3%) individuals were HAV seropositive without prior hepatitis A vaccination, thus indicating a previous HAV infection. Antibody prevalence (attributable to vaccination or infection) increased significantly with age. Multivariate logistic regression revealed that predominantly children and adolescents with migration background–even if they were born in Germany–are affected by HAV infections. Our results provide a rationale to emphasise existing vaccination recommendations and, moreover, to consider additional groups with a higher risk of infection for targeted vaccination, especially children with a migration background.

## Introduction

Hepatitis A is a vaccine-preventable disease caused by the hepatitis A virus (HAV) which is transmitted mainly via the faecal-oral route through close personal contact or contaminated food and water^1^.

The course of disease is variable, ranging from mild gastrointestinal symptoms to a severe icteric illness lasting several months. In general, symptoms are more severe in the elderly and people with weakened immune system. Particularly patients with chronic liver diseases are at risk of developing fulminant hepatitis A with a possible fatal outcome^2,3^.

Even though HAV infections in children often remain subclinical, children nevertheless have a key role in the epidemiology of hepatitis A. Firstly, particularly young children do not have strong individual hygiene skills, thus facilitating transmission of HAV via the faecal-oral route. Secondly, in countries without universal childhood vaccination and with low HAV endemicity, children are usually not immune and thus highly susceptible to HAV infection. Moreover, due to the relatively high infectivity of HAV together with a low rate of HAV immunity (lack of herd immunity), the outbreak potential in children is significant. Thirdly, HAV infections in children are mainly mild or even asymptomatic but are still quite common, so that numerous infections can go unnoticed which further promotes the spread of HAV^4–9^.

HAV endemicity is largely dependent on socio-economic development and typically highest in regions with poor sanitation and without access to safe drinking water^10–13^. In addition, travel abroad and (imported) contaminated food products are confirmed sources of infection^12,14,15^. Although Germany is regarded as a country with low HAV endemicity, two-thirds of the reported cases of hepatitis A are due to autochthonous transmission^16^.

Available hepatitis A virus vaccines are safe and highly effective and employed for routine childhood vaccination programs in some parts of the world^9,17–20^.

In Germany, the Standing Committee on Vaccination generally recommends vaccination for travellers to HAV-endemic areas but does not advocate universal childhood vaccination^21^.

Hence, elucidating factors associated with HAV infections, identifying vaccination gaps, and characterising groups at particular risk are important steps to further reduce HAV transmission.

To this end, we analysed HAV seroprevalence among German children and adolescents aged 3–17 years participating in a nationwide, population-based, cross-sectional survey and investigated associated demographic factors consecutively in vaccinated and infected individuals.

## Material and Methods

### Study design

Our study was performed within the framework of the German KiGGS survey (“German Health Interview and Examination Survey for Children and Adolescents”), a nationwide, population-based, cross-sectional survey, conducted between 2003 and 2006. In brief, a two-staged cluster-sampling was applied. At the first stage, 167 sample points (municipalities) were randomly selected across Germany, followed by the second stage with a random selection of 8–10 children or adolescents per age group and sample point from the population register, respectively^22,23^. Vaccination cards were requested from all participants. Serum samples (to test for anti-HAV antibodies) were available for participants aged 3 years and older. In order to be able to distinguish HAV seropositivity due to hepatitis A immunisation (>=1 shot of hepatitis A vaccine) and HAV infection, we focused on individuals having provided a vaccination card in addition to the serum sample. All other participants were excluded from the analysis. A weighting factor was introduced that corrects the sample’s deviations (sample with serum available) from the (German) population structure with regard to age, gender, region, nationality, type of municipality, and the education status of the head of the household.

### Quantification of anti-HAV antibodies

Each serum sample (among participants aged 3–17 years) collected within the framework of the German KiGGS survey was tested for total anti-HAV-antibody levels (IgM and IgG) by using an established quantitative immunoassay (Elecsys anti-HAV, Roche Diagnostics, Mannheim, Germany) according to the manufacturer’s instructions^24,25^. The detection range of the test is linear between titres of 3.0–60.0 IU/L. In accordance to the manufacturers’ instructions, sample titres <20.0 IU/L were classified as seronegative and titres >=20.0 IU/L seropositive, respectively. Of note, anti-HAV-IgG titres of 20.0 IU/L or above are commonly defined as protective^4,26–31^.

### Statistical analysis

Statistical analysis was performed using STATA 13 software (StataCorp LP, College Station, TX, USA). In order to account for the cluster structure of the sampling method, sampling weights were used in the statistical analysis to correct for disparities (of the subsample for which serum was available) from German population statistics.

We analysed the following outcome variables in three separate models: (previous) HAV infection, hepatitis A vaccination and overall-HAV seropositivity. Specifically, our analysis assessed the influence of the following explanatory variables in relation to all three aforementioned outcomes: age, gender, place of residence, population size of the community of residence, (parental) socio-economic status, and migrant status. The appropriate dataset was extracted from the self-reported questionnaires of the German KiGGS survey.

For each parameter, the prevalence and odds ratio (OR) (univariable logistic regression) was calculated with a 95% confidence interval (95% CI). When appropriate, the Wald test was used and a value of p < 0.05 was considered statistically significant. In logistic regression analyses, age (years) was used as continuous variable, all other variables were treated as categorical variables. The reference group was set to the lower or upper boundary for continuous or ordinal variables. For nominal variables, the reference group was set to the group having the lowest prevalence. For multivariable logistic regression, we started with a model including all parameters (full model) and excluded stepwise the least statistically significant parameter (backward elimination). Backward elimination was terminated after no parameter with a p-value of p > 0.05 remained in the stepwise-reduced model and the included parameters were considered to be statistically significant. For this purpose, an F-test was applied in order to compare different sub-models. Ultimately, all initially eliminated parameters were reintroduced separately into the final model, to test if they contribute meaningfully to the selected final model (F-test, p > 0.05).

### Definition of German regions of places of residence (consolidated federal states)

In our study, the place of residence of all 16 German federal states was consolidated into 6 areas: “North-West”, “Central-West”, “South-West”, “North-East”, “Central-East”, and “Berlin”, respectively.

North-West included the federal states Bremen, Hamburg, Lower Saxony, and Schleswig-Holstein, Central-West North Rhine-Westphalia, and Hesse, South-West Rhineland-Palatinate, Saarland, Baden-Wuerttemberg, and Bavaria, North-East Mecklenburg Western-Pomerania, and Brandenburg, and Central-East Saxony-Anhalt, Saxony, and Thuringia, respectively.

### Definition of categories for the population size of the community of residence

The population size of the community of residence was categorised into 4 sizes (“rural area” (<5,000), “small town” (5,000–<20,000), “medium-sized town” (20,000–<100,000), and “large town” with >=100,000 residents, respectively).

### Definition of categories for socio-economic status

Information on the socio-economic status was grouped into 3 categories: “low”, “medium”, and “high” according to Lange M. *et al*.^32^.

### Definition of categories for migration status

The migration status was categorised into “Non-migrant”, “One-sided”, and “Two-sided” migrant, respectively. For non-migrants none of the parents had immigrated to Germany. A one-sided migrant had one of the parents that immigrated to Germany. For a two-sided migrant the child itself and one of the parents immigrated or alternatively, both of the parents immigrated to Germany^33^.

### Ethics Statement and informed consent

This observational epidemiological study was approved by the Federal Office for Data Protection and by the ethics committee of Charité University Medicine, Berlin, Germany. Parents of the participants and all adolescents aged 14–17 years signed the written informed consent to the KiGGS study and the laboratory testing of their blood samples.

## Results

### Study response, and availability of vaccination cards and serum samples

**Figure 1:**
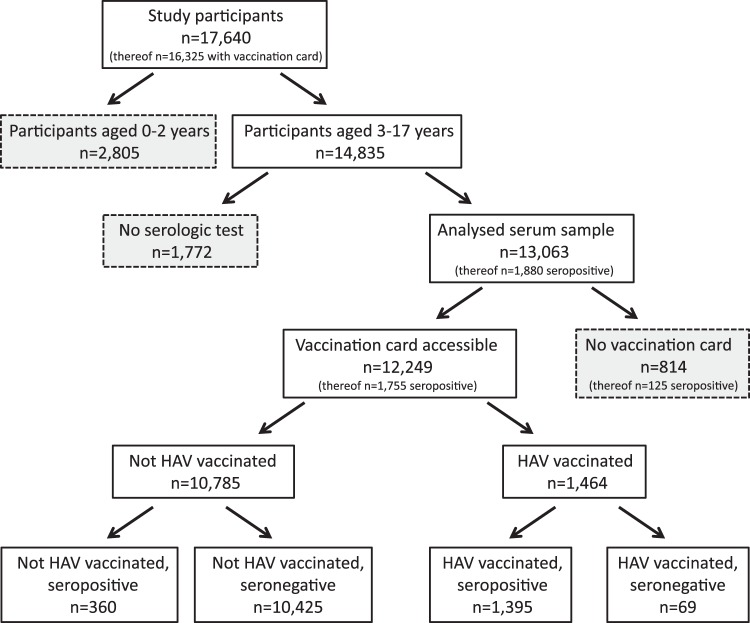
Flow chart and sample frequencies of participation by HAV serology and vaccination status.

Of 28,299 randomly selected study participants, 17,640 responded and also completed the provided questionnaire. The response rate of 66% and the analysis of non-response are detailed elsewhere^23^. Vaccination cards were available for 16,325 (93%) participants. HAV serology was intended only for study participants aged 3 years and older (n = 14,835) and among these, a total of 13,063 (88%) samples were tested for anti-HAV antibodies. Both, serum sample and vaccination card were available for 12,249 (82%) out of all 14,835 study participants aged 3–17 years (Fig. 1).

### Seroprevalence of anti-HAV antibodies in children and adolescents

Of 13,063 children and adolescents tested for anti-HAV antibodies, 1,880 (14%) were found to be seropositive (>=20 IU/L). A histogram depicting the distribution of the 13,063 analysed anti-HAV antibody test results is shown in the supplement (Supplementary Fig. S1).

**Figure 2:**
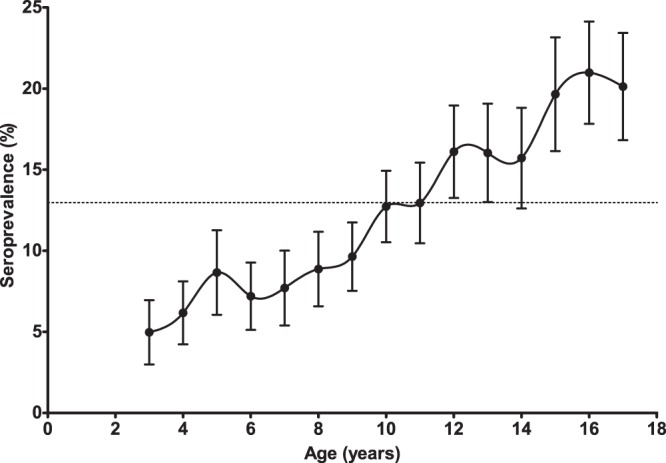
Estimated (weighted) proportion of HAV-seropositive study participants by age (error bars indicate 95% confidence intervals (95% CI), German children and adolescents, aged 3–17 years, n = 13,063). The dashed line depicts the estimated mean of the HAV-seroprevalence (13%). Serum samples of children aged 0–2 years were not requested. (The specific values per age including the respective 95% confidence intervals are provided in the supplement; Supplementary Table S1).

The weighted overall seroprevalence for anti-HAV antibodies in the German population aged 3–17 years was 13% (95% CI: 12–14%). Seroprevalence was strongly correlated with increasing age (Fig. 2, Supplementary Table S1). A significantly higher seroprevalence was observed in children or adolescents with a two-sided migration status as compared to non-migrants and one-sided migrants, respectively. In addition, seroprevalence differed at the regional level, with a notably high prevalence in Central-East Germany. Seroprevalence did not differ significantly by gender, community size and socio-economic status (Table 1).

**Table 1:** Overall HAV-seroprevalence: estimated (weighted) prevalence of HAV-specific antibodies in German children and adolescents (aged 3–17 years) by demographic characteristics and (weighted) results of univariable and multivariable analyses (n = 13,063).

Characteristic (total no.)	(weighted) seroprevalence	(weighted) univariable analysis		(weighted) multivariable analysis	
	% (95% CI)	Odds ratio (95% CI)	p-value	Odds ratio (95% CI)	p-value
Anti-HAV tested (13,063)	12.97 (11.76–14.29)	—	—	—	—
Age (yearly)	—	1.12 (1.10–1.13)	<0.001	1.11 (1.09–1.13)	<0.001
Sex					
male (6,708)	12.46 (11.09–13.97)	ref.	—	—	—
female (6,355)	13.52 (12.22–14.93)	1.10 (0.99–1.22)	0.085	—	—
Place of residence					
North-West (1,641)	7.49 (5.96–9.02)	ref.	—	ref.	—
Central-West (3,123)	10.32 (9.01–11.63)	1.42 (1.09–1.85)	0.009	1.36 (1.02–1.82)	0.034
South-West (3,912)	14.22 (11.85–16.60)	2.05 (1.53–2.75)	<0.001	2.08 (1.50–2.86)	<0.001
North-East (1,390)	14.02 (10.99–17.06)	2.01 (1.44–2.82)	<0.001	2.14 (1.48–3.10)	<0.001
Central-East (2,609)	25.74 (20.73–30.75)	4.28 (3.04–6.03)	<0.001	4.80 (3.33–6.93)	<0.001
Berlin (388)	14.31 (10.03–18.59)	2.06 (1.36–3.12)	0.001	1.81 (1.17–2.80)	0.008
Populationsize of municipality					
<5,000 (rural area) (2,875)	14.42 (10.03–18.08)	ref.	—	—	—
5,000–<20,0000 (small town) (3,470)	13.36 (10.83–15.89)	0.92 (0.60–1.39)	0.677	—	—
20,000–<100,0000 (medium-sized town) (3,778)	11.75 (9.95–13.55)	0.79 (0.53–1.17)	0.242	—	—
>100,000 (large town) (2,940)	12.94 (11.14–14.75)	0.88 (0.60–1.30)	0.528	—	—
Socio-economic status					
low (3,415)	12.43 (10.61–14.26)	ref.	—	ref.	—
medium (6,037)	11.85 (10.43–13.27)	0.95 (0.82–1.10)	0.462	1.02 (0.88–1.18)	0.795
high (3,292)	14.25 (12.45–16.05)	1.17 (0.98–1.40)	0.087	1.44 (1.20–1.74)	<0.001
unknown (319)	—	—	—	—	—
Migration status					
Non-migrant (10,203)	11.81 (10.38–13.24)	ref.	—	ref.	—
One-sided (879)	12.58 (9.85–15.31)	1.08 (0.83–1.40)	0.586	1.32 (1.01–1.73)	0.043
Two-sided (1,928)	18.24 (16.07–20.41)	1.67 (1.40–1.99)	<0.001	1.93 (1.57–2.36)	<0.001
unknown (53)	—	—	—	—	—

All study participants with an available HAV serology are included in the analysis (see Fig. 1).

In a weighted multivariable model, increasing age, having a high socio-economic status, a one-sided or a two-sided migration status, and living outside of North-West Germany was independently associated with HAV seropositivity (Table 1).

Seropositivity may have resulted either from HAV infection or hepatitis A vaccination. Of all 1,880 seropositive tested individuals 1,395 (74%) were vaccinated against hepatitis A, 360 (19%) were not vaccinated but seropositive, and 125 (7%) had an unknown vaccination status.

Accordingly, we also assessed HAV infections and hepatitis A vaccinations in separate analyses with a restriction to those 12,249 participants with both, available vaccination card and serum sample test result.

### HAV infections in children and adolescents

No hepatitis A vaccination was recorded in 10,785 (88%) of the 12,249 participants with available vaccination card and serum sample test result. However, 360 (3%) of the 10,785 unvaccinated participants were found to be seropositive, thus indicating a previous HAV infection. This corresponds to a weighted prevalence of HAV infection among the unvaccinated, anti-HAV antibody-tested children and adolescents of 3% (95% CI: 3–4%).

Increasing age correlated significantly with a higher prevalence of HAV infections in children and adolescents. In addition, the prevalence of HAV infection was particularly high in children or adolescents with a two-sided migration status, a low socio-economic status and (on the regional level) among those living in Berlin or Central-East Germany. The prevalence of HAV infection did not differ significantly by gender and community size (Table 2).

**Table 2:** HAV-infections: estimated (weighted) prevalence of HAV-specific antibodies due to HAV infection in German children and adolescents (aged 3–17 years) by demographic characteristics and (weighted) results of univariable and multivariable analyses (n = 10,785).

Characteristic (total no.)	(weighted) prevalence	(weighted) univariable analysis		(weighted) multivariable analysis	
	% (95% CI)	Odds ratio (95% CI)	p-value	Odds ratio (95% CI)	p-value
Anti-HAV tested and vaccination card available (10,785)	3.24 (2.87–3.66)	—	—	—	—
Age (yearly)	—	1.08 (1.04–1.11)	<0.001	1.07 (1.04–1.11)	<0.001
Sex					
male (5,552)	2.91 (2.45–3.46)	ref.	—	—	—
female (5,233)	3.59 (3.06–4.20)	1.24 (0.99–1.56)	0.065	—	—
Place of residence					
North-West (1,423)	2.42 (1.71–3.42)	ref.	—	ref.	—
Central-West (2,676)	3.63 (3.04–4.32)	1.51 (1.02–2.26)	0.042	1.31 (0.87–1.97)	0.193
South-West (3,228)	3.00 (2.32–3.74)	1.23 (0.80–1.89)	0.354	1.16 (0.75–1.80)	0.508
North-East (1,174)	2.67 (1.86–3.82)	1.10 (0.66–1.85)	0.704	1.38 (0.80–2.38)	0.242
Central-East (1,954)	4.32 (3.09–6.01)	1.82 (1.11–2.99)	0.019	2.30 (1.37–3.88)	0.002
Berlin (330)	5.03 (3.00–8.32)	2.13 (1.12–4.07)	0.021	1.76 (0.90–3.45)	0.097
Populationsize of municipality					
<5,000 (rural area) (2,382)	3.05 (2.24–4.13)	ref.	—	—	—
5,000–<20,0000 (small town) (2,880)	2.85 (2.10–3.85)	0.93 (0.60–1.45)	0.759	—	—
20,000–<100,0000 (medium-sized town) (3,147)	3.13 (2.63–3.73)	1.03 (0.72–1.48)	0.873	—	—
>100,000 (large town) (2,376)	3.95 (3.21–4.85)	1.31 (0.89–1.92)	0.165	—	—
Socio-economic status					
low (2,792)	4.18 (3.36–5.19)	ref.	—	—	—
medium (5,083)	2.68 (2.20–3.26)	0.63 (0.47–0.85)	0.002	—	—
high (2,708)	2.58 (2.04–3.25)	0.61 (0.44–0.85)	0.003	—	—
unknown (202)	—	—	—	—	—
Migration status					
Non-migrant (8,542)	2.28 (1.94–2.68)	ref.	—	ref.	—
One-sided (726)	2.97 (1.92–4.58)	1.31 (0.80–2.15)	0.281	1.45 (0.88–2.39)	0.148
Two-sided (1,479)	7.76 (6.34–9.48)	3.60 (2.76–4.70)	<0.001	3.84 (2.92–5.04)	<0.001
unknown (38)	—	—	—	—	—

The results refer to a subsample of study participants with HAV serology but who have not been vaccinated (see Fig. 1).

In a weighted multivariable model, increasing age, having a two-sided migration status, and living in Central-East Germany was independently associated with a higher prevalence of HAV infection (Table 2).

### Hepatitis A vaccinations in children and adolescents

Of 12,249 individuals with both, an available vaccination card and a test result for anti-HAV antibodies, 1,464 (12%) were vaccinated against hepatitis A (>=1 shot of hepatitis A vaccine). Most of the 1,464 individuals vaccinated against hepatitis A were found to be seropositive (1,395, 95%). However, a total of 69 (5%) were seronegative (<20 IU/L) in spite of having a documented vaccination history.

The weighted prevalence of hepatitis A vaccination in German children and adolescents was 11% (95% CI: 9–12%). Overall, the proportion of hepatitis A vaccinated individuals increased with age (p < 0.05). In addition, a significantly higher prevalence was observed in children or adolescents having a high or medium socio-economic status, respectively. Prevalence of hepatitis A vaccination differed at regional level with a notably high prevalence in Central-East Germany. Prevalence did not differ significantly by gender, community size and migration status (Table 3).

**Table 3:** Hepatitis A vaccination: estimated (weighted) prevalence of hepatitis A vaccination in German children and adolescents (aged 3–17 years) by demographic characteristics and (weighted) results of univariable and multivariable analysis (n = 12,249).

Characteristic (total no.)	(weighted) prevalence	(weighted) univariable analysis		(weighted) multivariable analysis	
	% (95% CI)	Odds ratio (95% CI)	p-value	Odds ratio (95% CI)	p-value
Vaccination card accessible (12,249)	10.51 (9.31–11.84)	—	—	—	—
Age (yearly)	—	1.13 (1.11–1.15)	<0.001	1.12 (1.09–1.13)	<0.001
Sex					
male (6,282)	10.30 (8.95–11.84)	ref.	—	—	—
female (5,967)	10.73 (9.51–12.08)	1.05 (0.94–1.17)	0.423	—	—
Place of residence					
North-West (1,503)	5.57 (4.34–7.12)	ref.	—	ref.	—
Central-West (2,877)	7.12 (5.98–8.46)	1.30 (0.94–1.80)	0.110	1.29 (0.94–1.79)	0.119
South-West (3,655)	12.08 (9.91–14.65)	2.33 (1.65–3.29)	<0.001	2.33 (1.63–3.33)	<0.001
North-East (1,335)	12.11 (9.06–16.00)	2.34 (1.54–3.55)	<0.001	2.11 (1.37–3.25)	0.001
Central-East (2,516)	23.66 (19.13–28.87)	5.26 (3.61–7.66)	<0.001	4.99 (3.38–7.39)	<0.001
Berlin (363)	8.95 (6.61–12.02)	1.67 (1.10–2.54)	0.017	1.66 (1.06–2.61)	0.028
Populationsize of municipality					
<5,000 (rural area) (2,763)	12.44 (8.16–16.72)	ref.	—	—	—
5,000–<20,0000 (small town) (3,298)	11.29 (8.79–13.79)	0.90 (0.56–1.43)	0.642	—	—
20,000–<100,0000 (medium-sized town) (3,505)	9.15 (7.44–10.86)	0.71 (0.45–1.10)	0.127	—	—
>100,000 (large town) (2,683)	9.78 (7.87–11.69)	0.76 (0.49–1.19)	0.235	—	—
Socio-economic status					
low (3,103)	8.56 (6.96–10.49)	ref.	—	ref.	—
medium (5,767)	10.26 (8.94–11.75)	1.22 (1.01–1.48)	0.044	1.16 (0.95–1.41)	0.149
high (3,156)	12.83 (11.11–14.77)	1.57 (1.24–1.99)	<0.001	1.67 (1.33–2.09)	<0.001
unknown (223)	—	—	—	—	—
Migration status					
Non-migrant (9,738)	10.64 (9.31–12.13)	ref.	—	—	—
One-sided (819)	11.07 (8.69–14.00)	1.05 (0.79–1.39)	0.754	—	—
Two-sided (1,649)	9.69 (7.92–11.81)	0.90 (0.72–1.13)	0.357	—	—
unknown (43)	—	—	—	—	—

The results refer to a subsample of study participants with HAV serology and accessible vaccination cards (see Fig. 1).

In a weighted multivariable model, increasing age, having a high socio-economic status, and living in South-West, North-East or Central-East Germany or Berlin, respectively, was independently associated with higher odds for hepatitis A vaccination (Table 3).

The analysis of aforementioned seronegative study participants (<20 IU/L) with a documented vaccination history (n = 69) revealed that 78% (n = 54) had been vaccinated with only 1 shot of hepatitis A vaccine (n = 22 monovalent, n = 27 bivalent vaccine, respectively and n = 5 vaccine unknown). In contrast, among the seropositive study participants (>=20 IU/L) with a documented vaccination history (n = 1395), 15% (n = 207) had been vaccinated with 1 shot of hepatitis A vaccine (n = 119 monovalent, n = 77 bivalent vaccine, respectively and n = 11 vaccine unknown).

## Discussion

The susceptibility to HAV infections is increasing in world regions with high socio-economic standards due to decreasing endemicity and the concomitant decline in naturally acquired immunity in childhood^15,34,35^. In order to provide a basis for an advanced prevention strategy, we quantified anti-HAV antibody levels in children and adolescents living in Germany and investigated epidemiological and socio-demographic aspects of HAV infections and hepatitis A vaccination.

In our nationwide, population-based, cross-sectional study, we demonstrated that 87% of German children and adolescents are susceptible to HAV. Moreover, we observed a significant age dependency, which reflects the cumulative life-time prevalence of HAV exposure or hepatitis A vaccination. These findings confirm the results of a previous report (Krumbholz *et al*.)^36^. Interestingly, in contrast to the aforementioned study (where males were found to have a significantly higher seroprevalence than females), we found a slightly higher seroprevalence in females instead of males, but this finding was not statistically significant (OR 1.10, 95% CI: 0.99–1.22). We suppose that this observed difference may have resulted due to the different study designs and study populations (nationwide, population-based, cross-sectional survey vs. sera taken from German paediatric centres).

Beyond that, our study also separately analysed seropositivity due to hepatitis A vaccination and HAV infection with regard to socio-demographic determinants. To our knowledge, this is the first nationwide, representative study conducted in industrialised countries which investigated both essential aspects of the epidemiology of hepatitis A using serological and vaccination data.

Overall, HAV immunity was mainly due to hepatitis A vaccination rather than HAV infection (11% vs. 3%). The low proportion of naturally acquired immunity in children and adolescents reflects a very low level of HAV endemicity in Germany^6,13,16,34^. The multivariable model indicated that two-sided migration status and living in Central-East Germany are independently associated with HAV infection. We assume this is probably due to different travel behaviour (to HAV endemic areas) and/or origin from HAV endemic countries. In this regard, particularly migrants may travel more frequently to regions with lower socio-economic standards that are HAV endemic (e.g. Turkey, Balkan countries) to visit friends and relatives or are visited by their relatives living in HAV endemic areas, here particularly asymptomatic children may start (unnoticed) transmission chains^4,37–41^. However, there may be other possible differences than traveling to HAV endemic areas that have not been investigated in our study.

In regard to hepatitis A vaccinations we found that regional differences as well as a higher socio-economic status were independently associated in the multivariable analysis. This finding could indicate that parents with a higher socio-economic status are better informed about the protective value of hepatitis A vaccination and therefore more frequently request immunisations for their children (e.g. possibly in connection with travel to HAV endemic regions). The marked difference with significantly higher hepatitis A vaccination in the region “Central-East” (with the federal states Saxony-Anhalt, Saxony, and Thuringia) can be explained by local vaccination recommendations in the federal state Saxony. In Saxony, the Federal Committee on Vaccination recommends (since 2002) either a complete regimen of HAV/HBV combination vaccinations for all children aged 2–17 years, or alternatively, following a complete HBV immunisation regimen in the first two years of life, a (single) HAV vaccination for children aged 2–17 years^42^.

Our study provides a snapshot of the situation surveyed ten years ago and we cannot exclude that the situation has changed in the past decade. However, we are assuming that possible changes would be rather modest (towards an even lower HAV seropositivity) and likely not vary the interpretation of demographic and epidemiological factors associated with the outcomes seropositivity, vaccination coverage and infection risks. In addition, we cannot preclude a potential cohort effect (e.g. specific travel preferences or the proportion and origin of children or adolescents born abroad). In order to reflect such possible changes (which may influence hepatitis A vaccination and HAV infection) a follow-up study would be useful to conduct in the years ahead.

In recent years, several food-borne outbreaks of hepatitis A have been described in Germany and other European countries^43–46^. These outbreaks affected mostly adults, possibly because children are more often asymptomatically infected. We assume that these incidents impacted the seroprevalence only marginally. However, we hope that such reports have been increasing awareness and resulted also in higher vaccination uptakes in children and adolescents.

Migration is another important factor to consider that may has been changing the seroprevalence of children and adolescents living in Germany. Particularly from 2015 to 2016 a larger influx of asylum seekers to Germany was reported^6^. Challenging travel conditions, and the high exposure risk related to poor hygienic standards when asylum seekers have fled to Germany, resulted in increased hepatitis A notifications. The increased hepatitis A notifications mainly related to children of asylum seekers shortly after arrival to Germany. Importantly, concerning German residents, no further transmission of HAV has been reported in this regard^6^. Assuming that a majority of asylum seekers successfully applied for asylum and is residing in Germany now, this influx would change the point estimate of a nationwide, population-based, cross-sectional survey. A recent study reported an anti-HAV antibody prevalence of 70% for this particular group (asylum seeking children <18 years with arrival to Germany in 2015)^47,48^. Although the prevalence is much higher than the observed overall seroprevalence in the German population aged 3–17 years (13%) it would only marginally increase the point estimate.

The observed overall response rate of 66% of the German KiGGS survey (“German Health Interview and Examination Survey for Children and Adolescents”) was in the range of comparable epidemiological studies^49^. To account for possible sampling bias and to ensure representativeness for children and adolescents living in Germany an analysis of non-response was conducted and a weighting factor was introduced in order to correct the sample’s deviations from the (German) population structure^22^.

The vaccination status in our study population was assessed using records in vaccination cards. Although this is a reasonable method, we are aware that those records occasionally might be incomplete. In such cases, this would have led to a misclassification and overestimation of HAV infected individuals. Such overestimation might be bypassed using specific serological methods (with HAV non-structural epitopes) which have been very recently described^50,51^. Thus, such a new technique could then also be implemented in a follow-up study.

In our study, we did not consider the completeness of the vaccination sequence among the study participants. Depending on the vaccine, two or more shots are recommended for a long-term protection against infections of HAV^52–55^. We found 69 (5%) participants being seronegative (anti-HAV-IgG titres of <20.0 IU/L) in spite of a documented vaccination history. The majority of these individuals had been vaccinated with one shot of hepatitis A vaccine only, underlining previous investigations that one dose of vaccine induces protective anti-HAV-IgG titres in approximately 95% of the vaccinees and only after a booster vaccination this proportion reaches 100%^56–62^.

In response to the steady decline in naturally acquired HAV immunity especially in children in many parts of the world, some countries have introduced universal childhood vaccination programmes in recent years^9,17–20,63,64^. The WHO suggests a vaccination strategy according to the HAV endemicity of the particular region^65–68^. In this context, for areas with a very low HAV endemicity, such as Germany, hepatitis A vaccination is currently recommended for high-risk groups only^34,69^.

The German Standing Committee on Vaccination recommends vaccination against hepatitis A for all individuals traveling to HAV endemic areas in addition to other risk groups such as men having sex with men (MSM) and persons with an occupational risks of HAV infection, respectively^21^. In Germany, travel-related vaccinations are not generally reimbursed. However, most German health insurance companies offer travel-related vaccinations as (free) incentives^70^.

The results of our study argue for targeting children and adolescents with a lower socio-economic status or a two-sided migration background more explicitly. Hence, health insurance companies, general practitioners and travel agencies among others could help to communicate the protection of hepatitis A vaccination to prospective travellers to endemic regions and recommend vaccination accordingly even (years) in advance. In addition, information should be provided on possible infection risks (particularly by asymptomatic children) during family visits to Germany from relatives living in HAV endemic areas.

As travel-related vaccinations are reimbursed by most German health insurance companies, not only information on the benefit of vaccination but also easier reimbursements could presumably increase vaccination coverage, especially in children and adolescents with a lower socio-economic status or a two-sided migration background.

Importantly, such a targeted vaccination policy would be beneficial not only for protecting this particular at-risk group, but would also reduce secondary infections and hepatitis A outbreaks in schools, kindergartens or day care facilities that may occur as a result of asymptomatic infected children or adolescents after returning from HAV endemic areas^37,38,40,71^.

In summary, our study reflects central aspects of the epidemiology of hepatitis A in German children and adolescents and provides arguments for a refined hepatitis A prevention strategy. Although the study was conducted in children and adolescents living in Germany, we believe many other low-incidence countries have a comparable epidemiological situation and our recommendation apply as well in these countries.

## Electronic supplementary material

Supplementary Information (Figure S1, Table S1)
