# Supplementary material for: Hepatitis A virus infections, immunisations and demographic determinants in children and adolescents, Germany
Source: Sci Rep. 2018 Nov 12;8:16696. doi: 10.1038/s41598-018-34927-1 (PMC6232152; doi:10.1038/s41598-018-34927-1)
Supplement: Supplementary file 1 — Supplementary Information (Figure S1, Table S1) [file 41598_2018_34927_MOESM1_ESM.pdf]

Hepatitis A virus infections, immunisations and demographic determinants in children and adolescents, Germany.

Kai Michaelis [1*], Christina Poethko-Müller [2], Ronny Kuhnert [2], Klaus Stark [1], and Mirko Faber [1]

[1] Department for Infectious Disease Epidemiology, Unit of Gastrointestinal Infections, Zoonoses, and Tropical Infections, Robert Koch Institute (RKI), D-13353 Berlin, Germany

[2] Department of Epidemiology and Health Monitoring, Robert Koch Institute (RKI), D-12101 Berlin, Germany

[*] Correspondence: K Michaelis

E-mail: MichaelisK@rki.de

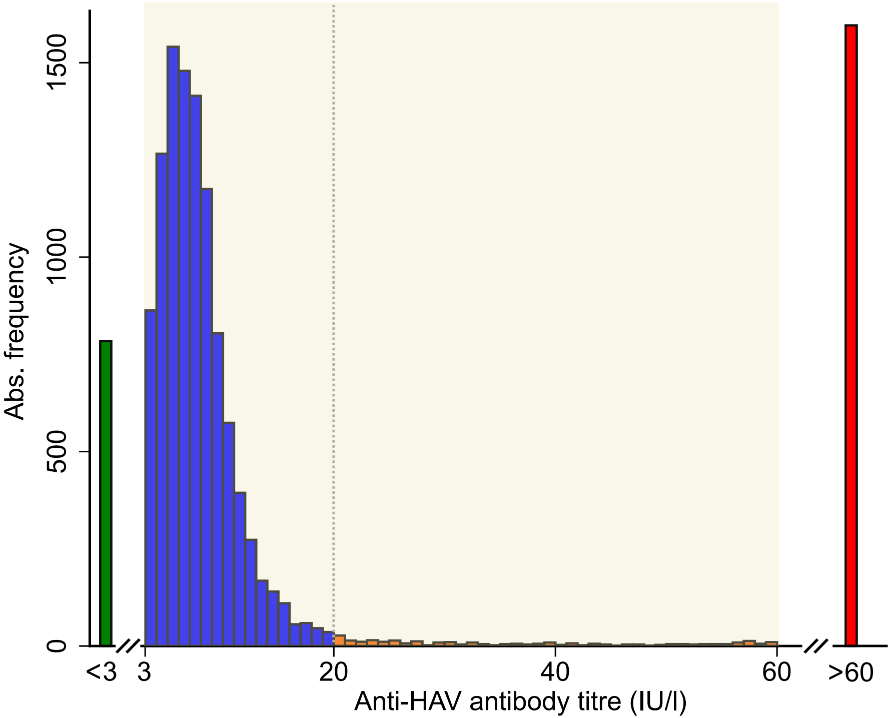

Abs. frequency
1500
1000
500
0
<3
3
20
40
60
>60
Anti-HAV antibody titre (IU/l)

Figure S1. Histogram of anti-HAV immunoassay test results in study participants (German children and adolescents, aged 3-17 years, n=13,063). The dashed line differentiates seronegative (<20.0 IU/L) from seropositive (>=20.0 IU/L) samples, respectively. The darker background depicts the linear detection range of the immunoassay (range 3-60 IU/L).

Of the 13,063 samples tested for anti-HAV antibodies 784 (6%) were seronegative and below the detection limit (<3 IU/L, green), 10,399 (80%) above the detection limit but also seronegative (range 3- <20 IU/L, blue), 284 (2%) seropositive, within linear detection range (20-60 IU/L, orange) and 1,596 (12%) seropositive, above the linear detection range (>60 IU/L, red) of the immunoassay, respectively (figure S1). Of all 1,880 seropositive tested samples 1,395 (74%) were vaccinated against hepatitis A, 360 (19%) were not vaccinated but seropositive, and 125 (7%) had an unknown vaccination status.

Table S1. Numerical values of the estimated (weighted) overall HAV-seroprevalence in German children and adolescents by age (n=13,063). These results are also depicted in figure 2 in the main text.

Age (years)	HAV-seropositive (%)	95% CI
0	n.d.	-
1	n.d.	-
2	n.d.	-
3	4.97	2.99-6.95
4	6.17	4.23-8.11
5	8.66	6.05-11.26
6	7.20	5.12-9.27
7	7.70	5.40-10.01
8	8.87	6.58-11.17
9	9.64	7.53-11.74
10	12.73	10.52-14.93
11	12.95	10.46-15.43
12	16.11	13.26-18.96
13	16.03	13.00-19.07
14	15.71	12.61-18.82
15	19.65	16.14-23.16
16	20.98	17.83-24.13
17	20.12	16.81-23.43
